# Place perception and restorative experience of recreationists in the natural environment of rural tourism

**DOI:** 10.3389/fpsyg.2024.1341956

**Published:** 2024-05-23

**Authors:** Ruomei Tang, Xinyu Zhao, Zixi Guo

**Affiliations:** ^1^College of Art & Design, Nanjing Forestry University, Nanjing, Jiangsu, China; ^2^Jinpu Research Institute, Nanjing Forestry University, Nanjing, Jiangsu, China; ^3^Digital Innovation Design Center, Nanjing Forestry University, Nanjing, Jiangsu, China

**Keywords:** rural environment, rural tourism, place perception, restorative experience, healthy environment

## Abstract

**Introduction:**

In contemporary society, people spend long periods under high stress, and tourism activities have gradually been internalized as a new means of stress release and self-recovery. Studies have found that the high-quality natural environment of rural tourism destinations has a higher restorative effect than other places, and the rural natural environment can provide psychological recovery to visitors on top of offering visual beauty and other experiences.

**Methods:**

This paper starts with the relationship between rural place perception and restorative experience evaluation. Based on theories such as the restorative environments theory, we investigates whether rural natural environmental factors have a restorative effect on recreationists through collecting 300 questionnaires and using SPSS 26.0 structural equation modeling for analysis.

**Results:**

The study found that there is a positive correlation between rural natural perception, place dependence, and restorative experience, forming a positive feedback loop dynamic system. The analysis suggests that enhancing the perception of the rural natural environment and place attachment can improve the restorative experience of recreationists in rural settings.

**Discussion:**

This research establishes a systematic research framework for the relationship between rural natural perception, place attachment, and restorative experience, to deeply understand the dynamic interaction between them. It reveals the relationship between rural natural perception and restorative experience, suggesting that enriching the perceptual elements in rural natural spaces can meet the diverse needs of recreationists, enhance the sense of dependence and identification with rural spaces, and thus promote the psychological well-being and restorative experience of recreationists. The study also finds that place dependence plays a mediating role between rural natural perception and restorative experience. Place attachment and place identity, as mediating variables, act as bridges and catalysts in the process of rural natural perception affecting restorative experience.

## 1 Introduction

Health-healing oriented tourism activities are increasingly becoming a proactive health practice in people’s lives, whereby travelers cross over into non-everyday environments through space, thus pursuing a mode of action for physical and mental restoration. Stress relief, physical and mental relaxation, and mindfulness have all become increasingly popular health and experience pursuits in tourism. According to Kaplan’s theory of attention restoration, the environment that can realize attention restoration has the characteristics of Being away, Soft fascination, Extent, and compatibility.w Some studies have proved that rural environment tourism is becoming more and more important as a resource to revitalize rural areas, has better development prospects, and that natural rural scenes have higher restorative effects than artificial scenes (Che et al., 2007; Li et al., 2022). Restorative factors in tourism environments are closely related to the participation of visiting tourists and the physical and mental health of local residents, and rural residents and tourists agree th0at rural natural landscape features are more popular than artificial landscape features in cities (Liu, 2011). Can the degree of perception of the natural environment increase the degree of restorative evaluation in a rural tourism environment? Place attachment is related to restorative perception, and nature perception can also influence the degree of place attachment to a tourist destination. Can the degree of nature perception be enough to positively influence restorative perception mediated by place attachment? These are all related to the restorative experience of rural tourism. Therefore, the relationship between rural nature perception and place attachment and restorative perception contributes to the development of rural restorative tourism experiences.

Most studies have focused on the relationship between rural tourism as a whole and restorative perception, or the comparison of restorative perception between rural natural environments and urban man-made environments, but there is a lack of research on the effects and relational mechanisms of place perception and restorative experience of rural environments on tourists. Moreover, existing studies mostly start from the single path of “place perception – restorative perception,” and there are fewer studies on the multi-dimensional path of rural natural environment perception, place attachment and restorative experience. Therefore, this paper focuses on the relationship between the degree of nature perception, place attachment and restorative evaluation of rural tourism, and takes the rural tourism environment of She Village in Nanjing City as the object of empirical analysis, and analyzes the positive correlation between the degree of tourists’ perception of the rural nature, the degree of place attachment, and the degree of restorative experience through the research and analysis of data and validation of the model and theories, and the positive correlation between the degree of tourists’ perception of the rural nature and the degree of place attachment and restorative experience, rural tourism environments created by favorable natural forms contribute to a restorative experience for tourists, with the aim of providing important conclusions to support the future transformation of rural development for wellness tourism.

## 2 Literature review

### 2.1 Restorative environment

Currently, many studies on the perception of restorative environments are based on the attention restoration theory proposed by Kaplan, to judge the restorative benefits of the environment based on the attention restoration theory, and summarize the restorative environment qualities into four qualities: Being away, Soft fascination, Extent and compatibility. By integrating the service scene model in tourism activities, some scholars have revealed the role of natural environmental elements in tourism service scenes in stimulating individual health and mental recovery from the natural dimension, which is also called “restorative scene.” Scholars have studied the restorative properties and qualities of different environments, and natural environments have more significant restorative effects than artificial environments such as urban and built environments (Herzog et al., 2003; Richardson et al., 2012), and proposed that natural healing landscapes should be created in natural wilderness spaces, and that good interactions with the landscapes should be carried out during the experience process to satisfy the needs of psychological relief and restoration of life balance (Kim et al., 2020). It is evident that places dominated by natural environments with restorative potential possess the ability to alleviate people’s attentional fatigue (Backman et al., 2023). Outdoor recreation and nature-based tourism experiences can provide spiritual restorative experience, which is considered to be the core restorative role of nature-based tourism (Qiu et al., 2021).

Research has proved that the natural environment has strong restorative benefits. The rural production and living environment are the most compatible with the great nature and has natural resource advantages. Studies on perception in rural environments in rural restorative environments have shown that some scholars believe that the textures and shapes in rural farmland, as well as the blue-green space and fresh air formed by abundant crops, can increase visitors’ visual preferences and the degree of restorative perception to a certain extent (Raman et al., 2021; Yang and Chen, 2021), and that restorative exercise in the natural environment of the countryside can also help individuals to have a restorative experience (Doughty, 2013; Kaley, 2017), examples of exercises are walking and jogging in the countryside. Thus, Rural tourism activities based on the natural environment help to restore sensory functions and improve the physical and mental health of tourists (Fritz and Sonnentag, 2006; Thompson et al., 2016).

In addition, through the establishment of research models, some scholars point out that the degree of restorative perception helps to improve the place attachment, health image and loyalty of tourism destinations, the healthy, organic and sustainable environment created by organic agritourism has restorative experience effect, which helps the transformation of rural tourism to health tourism (Xue and Shen, 2022). Meanwhile, as far as the local culture of the rural villages is concerned, some scholars have carried out certain explorations, taking the domestic Bama tourist villages as a case study, and found that the natural environment, the social environment and the symbolic landscape work together in the healing process of the tourists, under the influence of the longevity culture, the symbolic landscape plays a dominant role. Other scholars, based on cognitive appraisal theory and self-regulation theory, have built and verified models to reveal the complex pathways of tourists’ spatial perception of rural cultural memory, which can produce tourists’ restorative perceptions through the processes of situational participation and place attachment (Chang and Li, 2022). The above studies show that rural environments meet the four qualities of restorative environments, and to a certain extent, they can alleviate tourists’ stressful emotions.

In the process of tourism development, the combination of rural environment and health tourism has become more obvious, deriving tourism industries such as rural health tourism and recreation tourism, and the relationship between the countryside and the restorative environment has received more and more attention and emphasis from the academic community. A large number of literature studies have shown that the natural environment space in rural tourism, as the basis of tourism activities, influences the tourists’ choice of tourist sites and satisfaction. Some scholars have used the data from questionnaire research and in-depth interviews to construct structural equation models and rooted theory analysis to focus on whether tourism has a healing effect on individuals, or have used qualitative and quantitative research methods to focus on the staged characteristics of healing, the logic of generation, and the construction of practice, to systematically analyze the process of healing in tourism, the results and influencing factors, and to summarize and propose a mechanism for the formation of healing in tourism and analyzing framework. Different researchers have established models to validate and show that the level of rural tourism participation has a significant positive effect on the perception of restorative environment, tourist satisfaction and repeat intention, and the effect of environmental restoration emphasizes the intrinsic perceptions generated in the interaction between people and the environment, which can also be mapped to the level of human-social interaction (Jo et al., 2013; Zhang et al., 2023). The above experiments found that rural environments are associated with stress recovery. Therefore, further attention should be paid to the relationship between place perception and restorative experience of the rural environment.

### 2.2 Perception of place

The degree of naturalness of visitors in the rural environment and place attachment belong to two concepts of place perception, and the degree of naturalness is used to describe the degree of naturalization that the visitors perceive in the rural environment (Qiu et al., 2021). The level of “naturalness” is an important factor influencing recreationists’ aesthetic preferences, and the “naturalness” of the environment can influence the subjective preferences of recreationists, which in turn evokes their attachment. Williams’ study points out that place attachment is divided into two dimensions: place dependence and place identity; place dependence refers to whether the landscape, public facilities, and services in a place satisfy the needs of individuals. It is the functional nature of tourist’s attachment to the destination. And place identity is more in the emotional level of identity, is the individual based on values, attitudes, preferences and other concepts for the place to produce a sense of belonging or emotional attachment. Place attachment is an emotional bond between a person and a specific place (Santucci and Antonelli, 2004). According to Korpela’s research, it was found that individuals in places with higher levels of attachment were able to fully recover (Rollero and De Piccoli, 2010; Scannell and Gifford, 2017). Some scholars point out that the mechanisms of health promotion in environments are attributed to the effects of individuals’ behaviors and subjective emotions in their environments. Therefore, it is necessary to pay more attention to the subjective experience and feelings of individuals in the environment. For example, scholars constructed a model of restorative perceptual influences on park recreationists, which revealed the following influence routes: “green space landscape perceptual naturalness – environmental preference – place identity – environmental restorativeness.” Similarly, some scholars have explored the restorative assessment path of forest rehabilitation for visitors by constructing a structural relationship model between perceived naturalness of the landscape, place attachment, and the assessment of health benefits. Studying the relationship between landscape, experience and place attachment in organic agritourism, it was found that landscape, as a mediating factor, can influence tourists’ experience and directly affect their degree of place attachment. Environmental places with restorative qualities can generate restorative perceptions and experiences for recreationists, improve recreational value, and thus increase individual emotional connections to places (Mayfield, 2011).

### 2.3 Restorative experiences

Experience is derived from the Latin word “Experientia,” which originally means “experiment,” “try,” etc. The term “experience” is interpreted from the physiological and psychological level as the overall psychological feedback generated by interacting with information from the surrounding environment, using the body as a perceptual mediator. In the experience of mobility in the rural environment, the physical objects and the environment affect the individual through vision and perception, which in a sense can provide a certain material basis for the individual’s restorative experience. Most scholars believe that “recovery” is a process or a result, a process of physical and mental energy re-acquisition (Hartig et al., 2003), while other scholars, from a physiological perspective, believe that “recovery” is a process of restoration of a physiological stress state. Similarly, restorative experience is also a process or result, a process in which the individual regains the balance of energy, mind and body. Stephen Kaplan and Rachel Kaplan proposed four necessary stages of restorative experience: Clearing the mind, Cognitive quiet, Reflection, and Contemplation. Other scholars have analyzed the concept of restorative experience and found that previous research on restorative experience is characterized by three aspects: access, process, and outcome. And based on the analysis of the three-level coding of rooted theory, it was found that the restorative experience of tourists is divided into the four dimensions of relaxation experience, psychological distancing, purification experience, and renewal experience. These four dimensions are the progress of the current research results, so the evaluation of restorative experience for tourists in this study will be further experimented and summarized in these four dimensions (Zhai et al., 2023).

### 2.4 Research framework

**Figure 1:**
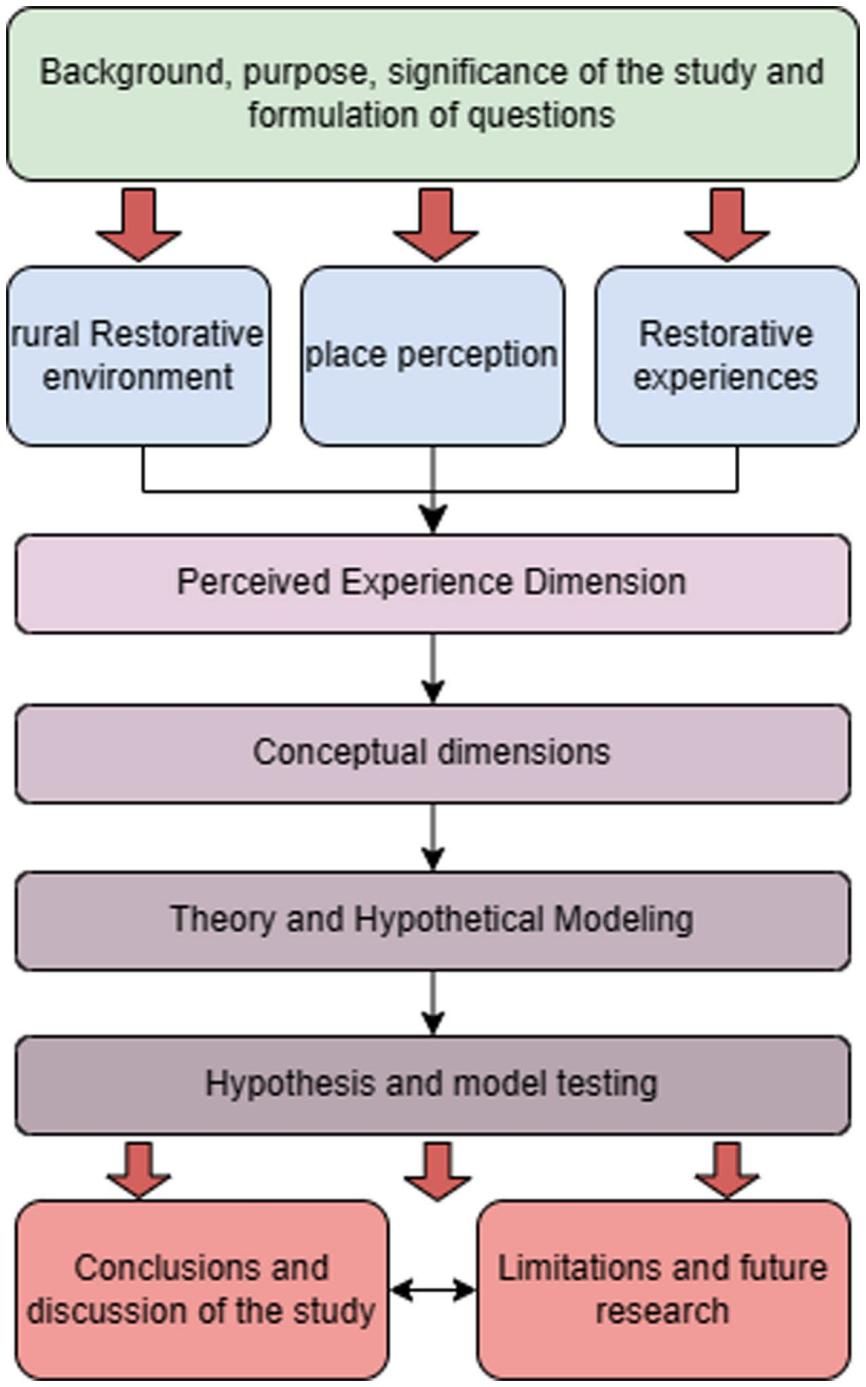
Research framework.

Tourists at this stage show a healthy pursuit of stress relief, physical and mental comfort, a positive state of mind, and individual well-being in rural tourism. It is therefore necessary for the study of rural tourism environments to re-conceptualize natural place perception, where rural natural restorative environments work to create a broader experience of wellness, and the following is a diagram of the logical framework for the study in this paper (Figure 1).

## 3 Research methods

### 3.1 Introduction of the study area

**Figure 2:**
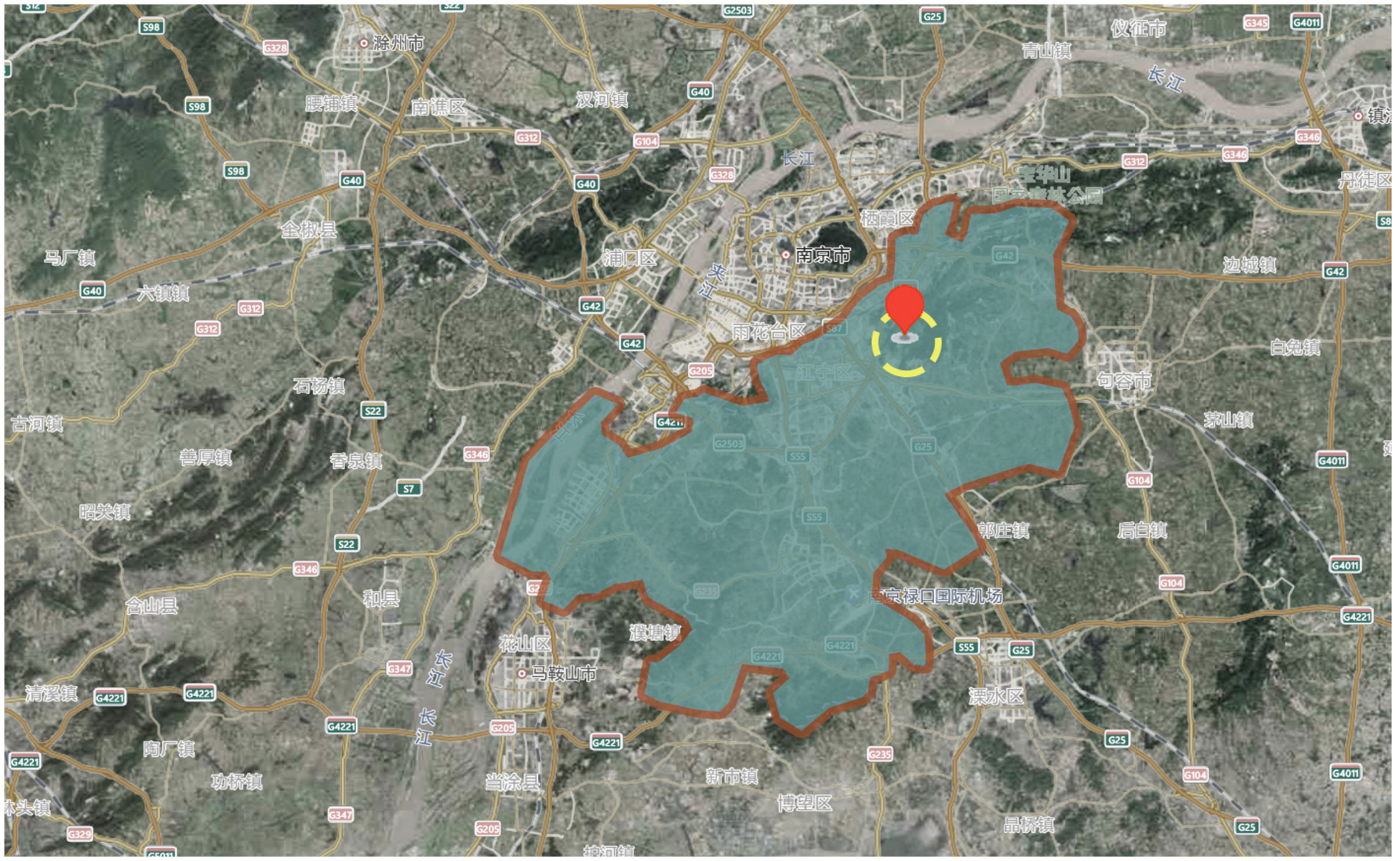
Location of the study area.

**Figure 3:**
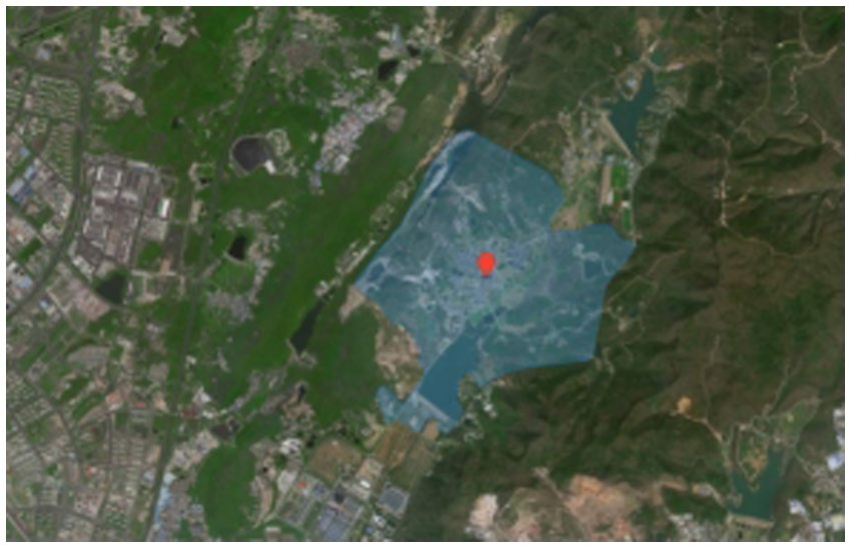
Scope of the study area.

**Figure 4:**
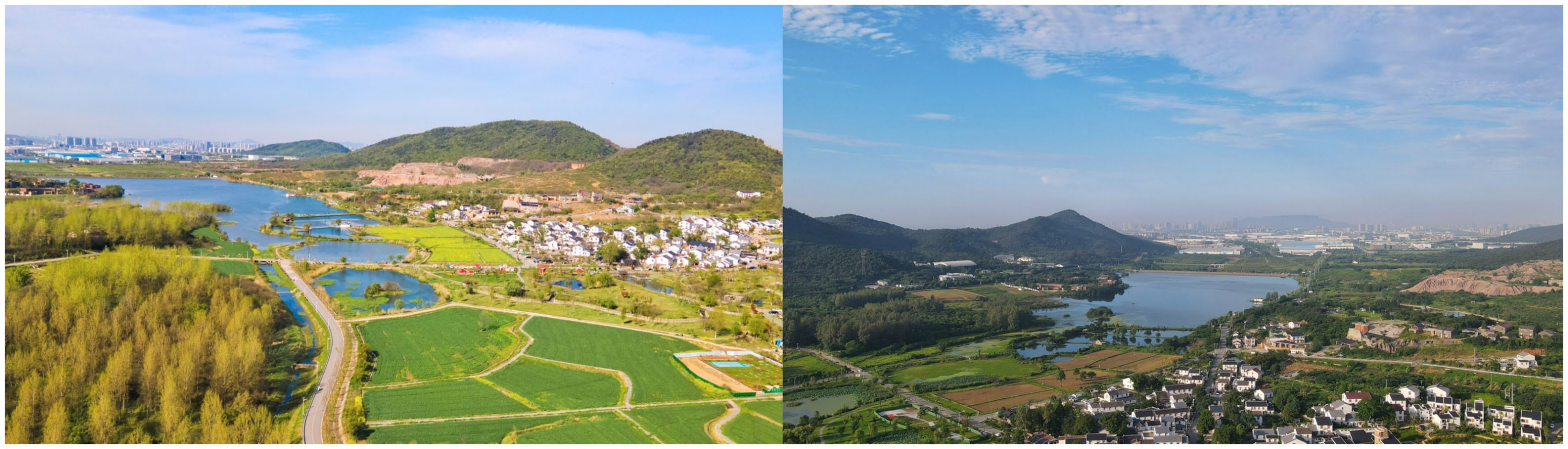
Status of the study site.

She Village is located in Shangxiao, Dongshan Street, Jiangning District, Nanjing City, Jiangsu Province, China, with a history of more than 600 years and a total area of 16.46 square kilometers (Figures 2, 3). She Village has a beautiful ecological environment and beautiful mountains. The highest peak is 275 meters above sea level; meanwhile, there are Shuanglong Lake and Tianyun Lake with a total water storage capacity of 4.39 million cubic meters. The houses in the village are of Ming and Qing Dynasty architectural styles and are arranged in a decent manner, with mountains, canyons, flat lakes, fields and villages merging into one, with colorful scenery; in addition, She Village has a mild climate, and grows rich crops as well as ornamental plants such as tea trees and seedlings. She Village is rich in produce, and the history and culture of She Village is profound, with well-preserved sites such as ancestral halls and monuments of Ming and Qing Dynasty buildings in the village (Figure 4).

In recent years, She Village increases the ecological environmental protection, promotes the construction of characteristic idyllic countryside, protects and inherits the history and culture of the ancient village, expands the scale of economic fruit forest planting, develops the tourism economy of flowers appreciation and fruits picking and develops a series of tourism projects such as Tianyun Lake Shuanglong Lake Reservoir Sightseeing, Mountain Bicycle Training Base, Lakeside Resort, Rainbow Road and Rafting etc., relying on the advantages of natural resources, and actively guides the farmers to operate farmhouse and lodging, enriching the business model of She Village, providing new content to the economy and industry, and devoting itself to the development of culture and tourism fusion industry. As a new development village in Nanjing, She Village is rich in village natural landscape, farmland resources and number of visiting tourists, and has formed a relatively stable rural natural landscape environment after a long period of time, which contribute to the measurement of place perception in rural environment. Therefore, the large number of visitors and abundant rural tourism landscapes in She Village are highly representative and typical for the study of place perception and restorative experience.

### 3.2 Study structure and hypotheses

Through literature analysis, we sorted out the influence mechanism between rural nature perception, place attachment and restorative experience, introduced arousal theory and natural regulation theory into the study of the relationship between the three, clarified the theoretical analysis framework and established modeling assumptions.

#### 3.2.1 The relationship between perceptual naturalness and place attachment in rural landscapes

Rural nature perception degree refers to the concept of “naturalness” in the forestry field, emphasizing the degree of natural landscape perceived by visitors in the rural environment, evaluating and quantifying the relationship between the degree of individual nature perception and place attachment, and distinguishing it from the concept of naturalness in ecology, the perception of the rural natural environment is the main driving factor influencing tourists’ behavioral preferences, place attachment and place identity. Naturalness in the environment has been proposed as a core dimension of place attachment and landscape preference, and research has confirmed that place attachment is positively correlated with perceived naturalness, and that respondents have a strong emotional connection to wild green spaces in their area (Colley and Craig, 2019). Scholars have constructed a landscape perception naturalness scale and validated it, and the results show that there are three dimensions of green space landscape perception naturalness: natural attribute perception, natural space perception, and natural form perception. Among them, natural attribute perception refers to the individual’s perception of the attributes of the rural natural environment, including biodiversity, vegetation coverage, sound diversity, etc.; natural spatial perception includes the contours and shapes of the natural environment as well as the artificial road environment, etc.; and natural morphology perception emphasizes the individual’s affective state and mentality response in the environment (Qunyi et al., 2017). The experimental study found that the naturalness of the tourist landscape has a significant positive effect on environmental preference, and there is also a significant positive relationship between environmental preference and place attachment. However, there may be variability in the results of structural stability evaluation in different research subjects and environments. In summary, the following hypotheses are proposed:

*H1*: There is a significant positive correlation between nature perception of rural landscapes and place dependence.

*H2*: There is a significant positive correlation between nature perception of rural landscapes on place identity.

#### 3.2.2 The relationship between place attachment and restorative experiences

Place attachment has been described by some scholars as a special emotional relationship that develops between a person and a place, with a tendency to show a willingness to stay in the place and a resulting psychological state of security and comfort, and also an integrated concept of person-psychological process-place interaction (Scannell and Gifford, 2010), which is considered to be divided into the two-dimensional place-dependence and place-identification (Williams and Vaske, 2003). Korpela and Hartig in their 1996 study argued that restorative environments can generate place attachment feelings in visitors (Santucci and Antonelli, 2004). The generation of place attachment is closely related to the environmental characteristics of the place, which include not only the physical and social environment, but also the symbolic environment. Some studies have shown that temporal, spatial, cultural, and emotional perceptions of rural cultural memory spaces are related to situational engagement, place attachment, and perceptual recovery. Traditional village visitors can be touched by traditional culture and memories in order to fulfill their emotional attachment to the local culture, thus achieving a restorative effect on the mind (Luo and Chiou, 2021). In addition, the social symbolic elements and natural elements of tourism experience scenes have significant positive effects on both place identity and place attachment; the physical elements have significant positive effects on place identity. However, at the same time, some scholars have found that there is no positive correlation between place attachment and restorative perception, while there is a significant positive correlation between place identity and restorative perception. In summary, this study proposes the following hypotheses:

*H3*: There is a significant positive correlation between place dependence on restorative experiences.

*H4*: There is a significant positive effect of place dependence on place identity.

*H5*: There is a significant positive correlation between place identity and restorative experience.

#### 3.2.3 The relationship between perceptions of rural nature and restorative experiences

The physical environment is extremely important in influencing the generation of local restorative, which is the fundamental condition for individuals to generate restorative experience and an important logical base map. The physical environment not only determines the siting of settlements, spatial patterns, architectural forms, etc. in the environment, but also determines the unique attitudes and lifestyles of local residents. According to the Attention Recovery Theory proposed by Kaplan, the physical environment has the characteristics of “remoteness,” “charm,” “complexity,” “compatibility” can realize individual attention recovery. The rural environment satisfies Kaplan’s characteristics of restorative environment in terms of geographic location, environmental atmosphere, and local customs.

Many scholars believe that there is a significant relationship between the individual’s perception of the natural landscape as well as the comfortable artificial environment in the countryside and the restorative experience produced by individual. Rural natural environments have the function of restoring people’s physiological and psychological health. Some research scholars point out that tourists are more likely to relax and increase their health perceptions of the vast fields, and argue that the essence of why tourism produces restorative experiences lies in the restorative quality of the tourism environment, suggesting that the restorative environment is the key to the restorative experience of tourists. In addition, some scholars have studied the degree of environmental restorativeness for different scenes in urban daily life and found that scenes with more natural elements have higher restorative perception scores (Herzog et al., 2003). The above studies suggest that the combination of natural landscape elements and good artificial elements in the countryside forms an environment with good landscape quality, which can promote the physical and mental health of individuals and make it easier for tourists to relieve their stress in the countryside environment. From this, it is inferred that the individual’s psychological recovery logic in the natural environment is: the natural landscape in the place – the individual’s place perception – resulting in a restorative experience. In the study of nature perception and restorative level, it was found that the elements of woodland, vegetable field, stream, and road in the rural environment had a significant positive effect on landscape preference and stress recovery, and based on the perceptual elements, the crowd could obtain a stronger stress recovery perception by strengthening the perception of the external rural environment such as the open view, serenity, and woodland. The degree of people’s restorativeness is closely related to the natural green space environment in the environment, and the closer the green space is to the natural landscape, the more restorative people are (Tveit et al., 2006; Akpinar, 2016).

At present, the restorative evaluation of the natural environment perception is mostly based on urban parks and forest rehabilitation as experimental objects, while the relationship between rural nature perception and restorative experience is less researched and lacks a corresponding evaluation index system. Therefore, this paper will propose evaluation indexes of rural nature perception based on existing research, and based on the data from the survey, conduct exploratory factor analysis, validation factor analysis and correlation analysis, etc., to construct a compatible index evaluation system.

*H6*: There is a significant positive correlation between rural nature perception and restorative experience

**Figure 5:**
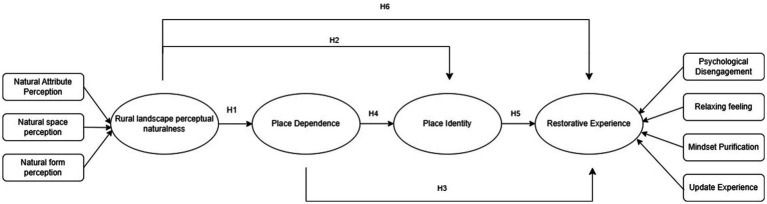
Research model diagram.

Thus, the research framework was established (Figure 5).

### 3.3 Questionnaire design

Using questionnaires as the main methodology, this study aims to conduct an empirical analysis using the Rural Environmental Recreationist Place Perception and Restorative Experience Assessment Model. The questionnaire scales used in the paper refer to existing well-established scales, including the Rural Nature Perception Scale, Place Attachment Scale and Restorative Experience Scale. Some other scholars have proposed concepts such as landscape naturalization including proportion of water bodies, proportion of vegetation, shape of vegetation, sense of wilderness experience, and management as indicators of nature perception (Tveit et al., 2006), as well as indicators such as landscape patchiness and continuity as judging criteria (Rechtman, 2013). Meanwhile, some scholars have also pointed out that rich biodiversity in rural landscapes can make people have stronger landscape preferences (Stokstad et al., 2016). Therefore, the biodiversity of the rural environment is also considered in the nature perception scale. In this study, exploratory factor analysis and correlation analysis were used in the research method. The specific steps are as follows: first, one-quarter of the overall questionnaire is set as the variable factor of landscape perception of naturalness, and the method of exploratory factor is applied to make the indicators in the scale effectively reflect the degree of individual perception of naturalness in the rural landscape; second, in order to ensure the differentiation of different interviewees and the homogeneity of the items’ measurements, the data analysis of the questionnaire is carried out by using SPSS26.0. The research scale in this paper includes four variable dimensions of rural nature perception, place dependence, place identity, and restorative experience, and each scale and measurement item have a corresponding theoretical basis. The details are shown in Table 1. Because there are relatively few studies on place perception and restorative aspects of rural environments, and because rural environments involve many elements of the natural environment, this study comprehensively refers to restorative research literature and research literature on rural and agricultural landscapes to determine the items. The final scale used in this study is shown in Table 2. All variables were measured on a 5-point Likert scale, where “1″ represents strongly disagree and “5″ represents strongly agree.

**Table 1:** Question items and sources for measuring perceived naturalness in rural landscapes.

Topics and sources			
Measured variable		Measurement item	Source of the title
A Rural landscape perceptual awareness	A1 Natural Property Perception	A11 More greenfield environments in the countryside	Williams and Vaske (2003), Li et al. (2017), and Geng et al. (2021)
		A12 More aquatic environments in the countryside	
		A13 More wildlife in the rural environment	
		A14 There are more natural sounds in the rural environment	
	A2 Natural Space Perception	A21 The natural environment in the countryside will have a sense of intimacy	
		A22 Natural spaces are quieter in the countryside	
		A23 You can get a sense of the wilderness experience in the countryside	
	A3 Natural form perception	A31 Varied contour lines of vegetation in the countryside	
		A32 Varied natural terrain in the countryside	
		A33 Water bodies in the countryside have a varied shape	
		A34 The roads in the countryside are more curvy	
B Attachment to place	B1 Territorial dependence	B11The natural environment of the countryside allows me to maximize my relaxation and leisure.	Williams and Vaske (2003)
		B12The natural environment of the countryside provides a better recreational experience than other environments	
		B13I like the nature of the countryside better than any other place.	
		B14My need for relaxation is best met in the natural surroundings of the countryside.	
	B2 Local identity	B21The natural environment of the countryside has a special meaning and deep affection for me.	
		B22I have enriched my understanding of myself in the natural environment of the countryside	
		B23I identify very much with the nature of the countryside	
		B24I’m very fond of the nature of the countryside	
C Restorative experiences	Psychological distancing Relaxing experience Purification Experience Updating experience	C12 It’s a place where I completely forget about everyday life.	Howard et al. (1993) and Sonnentag and Fritz (2007)
		C13 This is where I release my stress and balance my mind and body.	
		C14 I feel relaxed and free here.	
		C15 Here my thoughts are organized	Liu et al. (2011)
		C16 I feel more at peace here.	
		C17 I feel like a new person here.	Howard et al. (1993) and Kim et al. (2012)
		C18 I feel energized here.	

**Table 2:** Sample reliability analysis.

Total item statistics				
Variable	Measurement options	Clone Bach after deleting item Alpha	Cronhach’s α	CR
A1	A11	0.682	0.747	0.73
	A12	0.691		
	A13	0.695		
	A14	0.686		
A2	A21	0.614	0.727	0.73
	A22	0.601		
	A23	0.698		
A3	A31	0.722	0.781	0.73
	A32	0.744		
	A33	0.735		
	A34	0.71		
B1	B11	0.697	0.73	0.78
	B12	0.652		
	B13	0.649		
	B14	0.678		
B2	B21	0.765	0.818	
	B22	0.794		
	B23	0.766		
	B24	0.756		
C1	C11	0.869	0.884	0.83
	C12	0.868		
	C13	0.872		
	C14	0.867		
	C15	0.87		
	C16	0.867		
	C17	0.872		
	C18	0.868		

### 3.4 Questionnaire investigation

The research data in this article was obtained through a questionnaire survey. The questionnaire survey was conducted on tourists from She Village, Nanjing City from May 28, 2023 to June 15, 2023. The questionnaire was filled out online and offline. The weather conditions were guaranteed to be good when distributed. Finally, 310 questionnaires were collected, and after excluding 10 invalid questionnaires, the effective questionnaire rate was 96.77%, meets the sample number of the questionnaire.

## 4 Results

### 4.1 Sample descriptive statistical analysis

Descriptive analysis of survey questionnaire samples (Table 3). In the surveyed samples, women and men each accounted for 50%. The sample population aged 31 to 40 is the largest, accounting for 30%, followed by the sample population aged 21 to 30, accounting for 28%; In terms of education level, the samples from vocational colleges and undergraduate universities have the highest proportion, accounting for 32 and 27% respectively; In terms of occupation, workers, business personnel, and service industries account for the largest proportion of the sample, accounting for 26%, followed by students accounting for 18%; Among the visitors to She Village, the largest proportion is foreign tourists, accounting for 69%, followed by local tourists, accounting for 19%.

**Table 3:** Descriptive features of the sample.

Frequency analysis of demographic variables					
Variable	Option	Frequency	Percentage	Average value	Standard deviation
Gender	A. Male	150	50%	1.50	0.50
	B. Female	150	50%		
Age	A. Under 20 years old	5	2%	3.25	1.09
	B.21–30	83	28%		
	C.31–40	91	30%		
	D.41–50	79	26%		
	E.51–60	37	12%		
	F. Over 60 years old	5	2%		
Education level	A. High school or below	68	23%	2.42	1.04
	B. Junior college education	95	32%		
	C. Degree	80	27%		
	D. Graduate or above	57	19%		
Career	A. Party and government officials	25	8%	4.14	1.99
	B. Individual and private owners	43	14%		
	C. Workers, business, service personnel	79	26%		
	D. Cultural and educational workers	32	11%		
	E. Technology and healthcare workers	13	4%		
	F. Student	54	18%		
	G. Other	54	18%		
Identity	A. Visiting tourists from other places	206	69%	1.46	0.77
	B. Local tourists	58	19%		
	C. Surrounding residents	28	9%		
	D. She cun staff	8	3%		

Based on the results of the above analysis, it can be seen that the numerical characteristics of the demographic variables reflect the distribution of the respondents in this survey. Where the mean value represents the concentration trend. The standard deviation represents the fluctuation. According to the results of frequency analysis of each variable, it can be seen that the distribution basically meets the requirements of the sample survey.

### 4.2 Reliability analysis

Exploratory factor analysis and reliability testing were conducted on 27 measurement items of the overall questionnaire (Table 4), and the results showed that the KMO value was 0.959, greater than 0.7. The coefficient range of the KMO test was between 0 and 1, and the closer it was to 1, the better the validity of the questionnaire. According to the significance of the spherical test, it can also be seen that the significance of this test is infinitely close to 0. Refusing the original hypothesis, the questionnaire has good validity.

**Table 4:** Sample significance results.

KMO Bartlett’s test		
KMO sampling suitability quantity		0.959
Bartlett sphericity test	Approximate chi square	3874.171
	Freedom	351
	Significance	0.000

In this study, the reliability analysis of the relevant variables was conducted. Guilford (1950) argued that the coefficient of Cronhach’s α is higher than 0.7, the consistency and reliability of the scale data is a high degree (Stroud, 1951). Based on the results of the above analysis, it can be seen that the Cronhach’s α between rural nature perception, place attachment and restorative experience are all higher than 0.7, and the combined Cronhach’s α in content is 0.948, which is higher than 0.7. Based on the reliability coefficients of the item deletion, it can be seen that all of them are less than the Cronhach’s α coefficients of the items. Therefore, the topics of the content dimension of place perception and restorative experience in rural environments do not need to be adjusted.

### 4.3 Correlation analysis

According to the above correlation analysis results (Table 5), there is a significant correlation between each variable, and the correlation coefficient is greater than 0, so there is a positive correlation between each variable. There is a close correlation between restorative experience and various dimensions of rural landscape perception naturalness, place dependence, and place identity, and the correlation coefficient is mostly above 0.6.

**Table 5:** Sample correlation analysis.

Correlation analysis between various dimensions							
Variable	Correlation	Natural attribute perception	Natural spatial perception	Natural form perception	Place dependence	Place identification	Restorative experience
Natural attribute perception	Pearson correlation	1					
Natural spatial perception	Pearson correlation	0.643**	1				
Natural form perception	Pearson correlation	0.692**	0.0.0.678**	1			
place dependence	Pearson correlation	0.605**	0.623**	0.635**	1		
Place identification	Pearson correlation	0.577**	0.553**	0.592**	0.721**	1	
Restorative experience	Pearson correlation	0.629**	0.601**	0.649**	0.767**	0.761**	1

**At the 0.01 level (double tailed), the correlation is significant.

### 4.4 Model results

**Figure 6:**
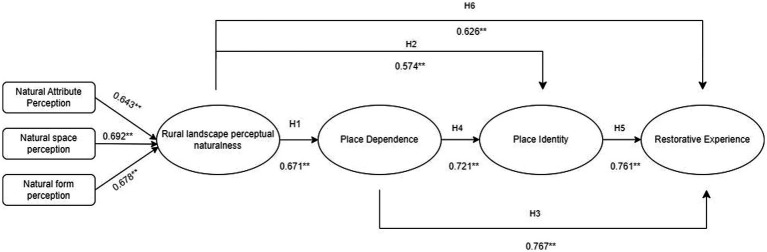
Correlation model.

The results showed that there was a significant positive correlation between rural nature perception on both place attachment and restorative experience, and the correlation between place dependence and place identity on restorative experience was the most significant, which was 0.767 and 0.761. Simultaneously, there was a significant positive correlation between rural nature perception and restorative experience, which was 0.626; and there was a significant positive correlation between rural nature perception and place dependence and place identity showed significant positive correlations of 0.671 and 0.574. Therefore, the hypotheses between H1–H6 were verified to be valid, and all of them were significantly positively correlated (Figure 6).

First, the degree of rural nature perception has a direct positive driving effect on restorative experience. From the results, the natural attribute perception, natural space perception, and natural form perception dimensions all have a significant positive effect on restorative experience, showing a significant correlation of 0.626. This is consistent with past research, indicating that the emotional connection formed between individuals and the natural environment in rural natural environments can help individuals obtain restorative experiences in that environment, and the stronger the environmental perception, the better the restorative experiences obtained.

Secondly, through the data analysis of place dependence and place identity, it was found that they also have a significant positive relationship with restorative experience, which is a significant correlation of 0.767 and 0.761 in the results. People’s sense of psychological dependence and identity generated in the place environment can directly affect the degree of restorative experience. The enhancement of the sense of dependence and identity of the place in rural space is beneficial to promote the perception of restorative benefits such as psychological distance, relaxation experience, spiritual purification, and renewal experience. Research has shown that the higher the dependence and sense of identity on the rural environment, the better the evaluation, the more conducive to the stimulation of people’s positive state, so that they can get rid of the negative physical and mental state, and the degree of physical and mental recovery is also increased.

Finally, place attachment acted as a mediating variable between rural nature perception and restorative experience, and place dependence produced a significant correlation coefficient of 0.721 on place identity. This suggests that rural nature perceptions can have a direct effect on restorative experiences and an indirect effect on restorative experiences through the mediating effects of place dependence and place identity. People’s perception of rural nature indirectly drives restorative experience through place dependence and place identity. The higher the recreationist’s attachment emotion to the rural environment, the more effective the restorative experience in that rural environment. Therefore, positive place attachment emotion building contributes to the perception of restorative experience in the population.

## 5 Discussion

Rural nature perception, place attachment, and restorative experience are all important concepts in the study of rural environments. This study aims to develop a systematic research framework based on the relationships among the three to help gain a deeper understanding of the dynamic interactions among these concepts. This includes the transformation from natural perception of the countryside to place attachment, the facilitating effect of place attachment on restorative experience, and the fact that restorative experience can in turn enhance natural perception of and place attachment to the rural environment, leading to a positive feedback loop. Existing studies basically focus on the influence of place environment on place attachment or on restorative experience, reflecting a point-to-point single-type research path and lacking system integration. This study emphasizes the complex and dynamic relationship between rural nature perception, place attachment, and restorative experience. It not only reveals the direct connection between these elements, but also highlights the interactions and feedback mechanisms among the three. This system of research allows for a more comprehensive understanding of the dynamic relationship between rural nature perception, place attachment, and restorative experience, and emphasizes the importance of considering nature perception and place attachment in rural planning and management to promote restorative experience.

This study aims to elucidate the relationship between rural natural perception and restorative experiences, demonstrating how individuals achieve psychological restoration through perceiving various aspects of the natural environment. This finding is in line with the biophilia hypothesis, which posits that the natural environment is a direct source of pleasure and health for humans. Additionally, the stress reduction theory suggests that the natural environment has a direct restorative effect on both body and mind. Moreover, this study emphasizes that rural natural perception is also one of the core dimensions of sense of place attachment, positively influencing individuals’ emotions through direct sensory experiences of the natural environment. Based on existing literature and research, this study focuses on three dimensions of rural natural perception: perception of natural attributes, natural spatial perception, and perception of natural forms. In the relationship between rural natural perception and restorative experiences, these three perceptual dimensions interact with each other, collectively promoting the individual’s restoration process. The overall perception of the rural environment by individuals is not only formed through singular sensory experiences but also through comprehensive perception of various aspects of the environment. The research indicates that diverse natural landscapes contribute to stress reduction and enhance psychological well-being. Well-designed and easily accessible natural spaces allow visitors to interact intimately with nature, facilitating psychological relaxation and restoration, thus enhancing restorative experiences. Suitable natural forms provide visual and sensory pleasure, promoting the psychological restoration process for individuals as they offer a means of escaping the everyday environment, aiding in mental recovery. The empirical results validate the rationality and effectiveness of the three-dimensional assessment of natural perception in this study, demonstrating accuracy in evaluating sense of place attachment and restorative experiences. Therefore, in the optimization process of rural tourism environments, with a focus on the restorative experiences offered by rural settings, it is essential to highlight the factors contributing to environmental restoration, enhance rural natural perception, and not only pay attention to individual environmental features but also consider how these features can work together to create an environment conducive to psychological restoration. While preserving rural characteristics, it is important to improve various public facilities. By magnifying and leveraging the ecological resources and natural attributes within rural areas, such as enriching agricultural landscapes, different types of recreational experiences for visitors can be enhanced in agritourism. By shaping the environmental image through the five senses of sight, hearing, touch, smell, and taste, the rural environment’s Being away, Soft fascination, Extent and compatibility should be emphasized as much as possible, to avoid visitors experiencing directional attention fatigue. Properly releasing stress in the rural natural environment allows for self-restoration.

This study found that sense of place attachment mediates the relationship between rural natural perception and restorative experiences. Place dependence, acting as a mediator variable, plays a bridging and catalyzing role in the process through which rural natural perception influences restorative experiences. Rural natural perception initially enhances both place dependence and place identity, which in turn enhance individual restorative experiences. This mediation process reveals a continuous pathway from rural natural perception to restorative experiences, with place dependence and place identity acting as bridges between the two (Scannell and Gifford, 2010) proposed a tripartite model of place attachment, which includes interactions between people, processes, and places, providing a detailed analysis of the formation process and mechanisms of place attachment (Raymond et al., 2010) studied how place dependence and place identity are associated with environmental values and behavioral intentions, emphasizing the importance of place attachment in environmental management. Building upon existing research, this study integrates theoretical frameworks from environmental psychology and place attachment research, focusing more on the interactive perspective between individual visitors and rural environments. It underscores the importance of emotional and cognitive processes of individuals toward rural environments in shaping restorative experiences, highlighting the role of emotional connections and identity in facilitating psychological restoration processes, providing a deeper understanding of restorative experiences. This contributes to a more comprehensive understanding of how rural environments promote psychological restoration and how designing and managing rural environments to enhance place dependence and place identity can improve the benefits of restorative experiences. From the perspective of place dependence, efforts should be made to increase and improve rural recreational facilities, strengthen service management, and meet the needs of different types of visitors, thereby enhancing functional attachment to rural environments. From the perspective of place identity, rural environments should reflect more regional elements and historical culture. Local rural culture is the vitality of rural sustainable development and serves as spatial imaginary symbols formed by people for the environment, symbolizing ideological and value systems. Enhancing rural environments based on both physical and social environments can effectively meet the emotional identification needs of visitors, generating more effective restorative experiences.

In this study, there are several shortcomings, primarily focusing on the relationship between rural environmental natural perception, sense of place attachment, and restorative experiences. Consequently, there was no examination of the functional facilities within rural environments, the emotional atmosphere they create, and their relationship with restorative experiences. Additionally, the study did not explore the characteristics of different visitor groups as a whole, which should be considered in future research to comprehensively understand the impact of rural environments on individual psychological restoration.

## 6 Conclusion

In conclusion, the study explored the dynamic relationship of the positive feedback loop existing between rural natural perception, sense of place attachment, and restorative experiences. The results indicate that rural natural perception has a positive driving effect on restorative experiences. Similarly, there is a significant positive relationship between sense of place attachment and restorative experiences. Acting as a mediator variable, sense of place attachment strengthens the connection between rural natural perception and restorative experiences. Rural natural perception can directly influence restorative experiences and can also indirectly affect them through the mediating effect of sense of place attachment. Focusing on the restorative experiences offered by rural environments, it is essential to emphasize the factors contributing to environmental restoration. By enriching the perceptual elements in rural natural spaces to meet the diverse needs of visitors, enhancing the sense of dependence and identity toward rural spatial places, one can promote the psychological well-being and restoration of visitors.

## Data availability statement

The original contributions presented in the study are included in the article/supplementary material, further inquiries can be directed to the corresponding author.

## Author contributions

RT: Writing – review & editing, Writing – original draft. XZ: Writing – original draft, Methodology, Investigation, Formal analysis, Data curation, Conceptualization. ZG: Writing – review & editing, Investigation, Validation.
